# The megamouth shark, *Megachasma pelagios*, is not a luminous species

**DOI:** 10.1371/journal.pone.0242196

**Published:** 2020-11-25

**Authors:** Laurent Duchatelet, Victoria C. Moris, Taketeru Tomita, Jacques Mahillon, Keiichi Sato, Catherine Behets, Jérôme Mallefet

**Affiliations:** 1 Marine Biology Laboratory, Earth and Life Institute, Université Catholique de Louvain, Louvain-la-Neuve, Belgium; 2 Okinawa Churaumi Aquarium, Motobu-cho, Okinawa, Japan; 3 Zoological Laboratory, Okinawa Churashima Research Center, Motobu-cho, Okinawa, Japan; 4 Laboratory of Food and Environmental Microbiology, Earth and Life Institute, Université Catholique de Louvain, Louvain-la-Neuve, Belgium; 5 Institut de Recherche Expérimentale et Clinique, Pôle de Morphologie, Université Catholique de Louvain, Woluwe-Saint-Lambert, Belgium; Laboratoire de Biologie du Développement de Villefranche-sur-Mer, FRANCE

## Abstract

Despite its five meters length, the megamouth shark (*Megachasma pelagios* Taylor, Compagno & Struhsaker, 1983) is one of the rarest big sharks known in the world (117 specimens observed and documented so far). This filter-feeding shark has been assumed to be a luminous species, using its species-specific white band to produce bioluminescence as a lure trap. Another hypothesis was the use of the white band reflectivity to attract prey or for social recognition purposes. However, no histological study has ever been performed to confirm these assumptions so far. Two hypotheses about the megamouth shark's luminescence arose: firstly, the light emission may be intrinsically or extrinsically produced by specific light organs (photophores) located either on the upper jaw white band or inside the mouth; secondly, the luminous appearance might be a consequence of the reflection of prey luminescence on the white band during feeding events. Aims of the study were to test these hypotheses by highlighting the potential presence of specific photophores responsible for bioluminescence and to reveal and analyze the presence of specialized light-reflective structures in and around the mouth of the shark. By using different histological approaches (histological sections, fluorescent *in situ* hybridization, scanning electron microscopy) and spectrophotometry, this study allows to unravel these hypotheses and strongly supports that the megamouth shark does not emit bioluminescence, but might rather reflect the light produced by bioluminescent planktonic preys, thanks to the denticles of the white band.

## Introduction

One rare and mysterious shark species, the megamouth shark, *Megachasma pelagios*, described in 1983 (Taylor, Compagno & Struhsaker) is assumed to display light emission capabilities [1–3]. Despite its length of over 5.5 m, only 117 specimens have been encountered across the oceans and only one specimen has been recorded up to now in its natural environment [4–6]. Like the basking shark, *Cetorhinus maximus*, and whale shark, *Rhincodon typus*, this sole member of the Megachasmidae (Lamniforme) is a filter shark feeding on plankton such as epi- and mesopelagic euphausiid shrimps (*Thysanopoda pectinata*, *Euphausia pacifica*), copepods, and sea jellies [7, 8]. Through vertical migration, the megamouth shark is assumed to follow zooplankton, as it is encountered in shallow waters during the night, and in deep waters during the day [2, 9]. Unlike the other filter-feeding sharks, *M*. *pelagios* seems to possess a unique feeding method likely derived from the ram-filter mode used by the basking shark [10, 11]. By creating a negative pressure when it expands maximally the oral cavity, it fills it and expulses the water through its gill slits when it closes it [3].

**Fig 1:**
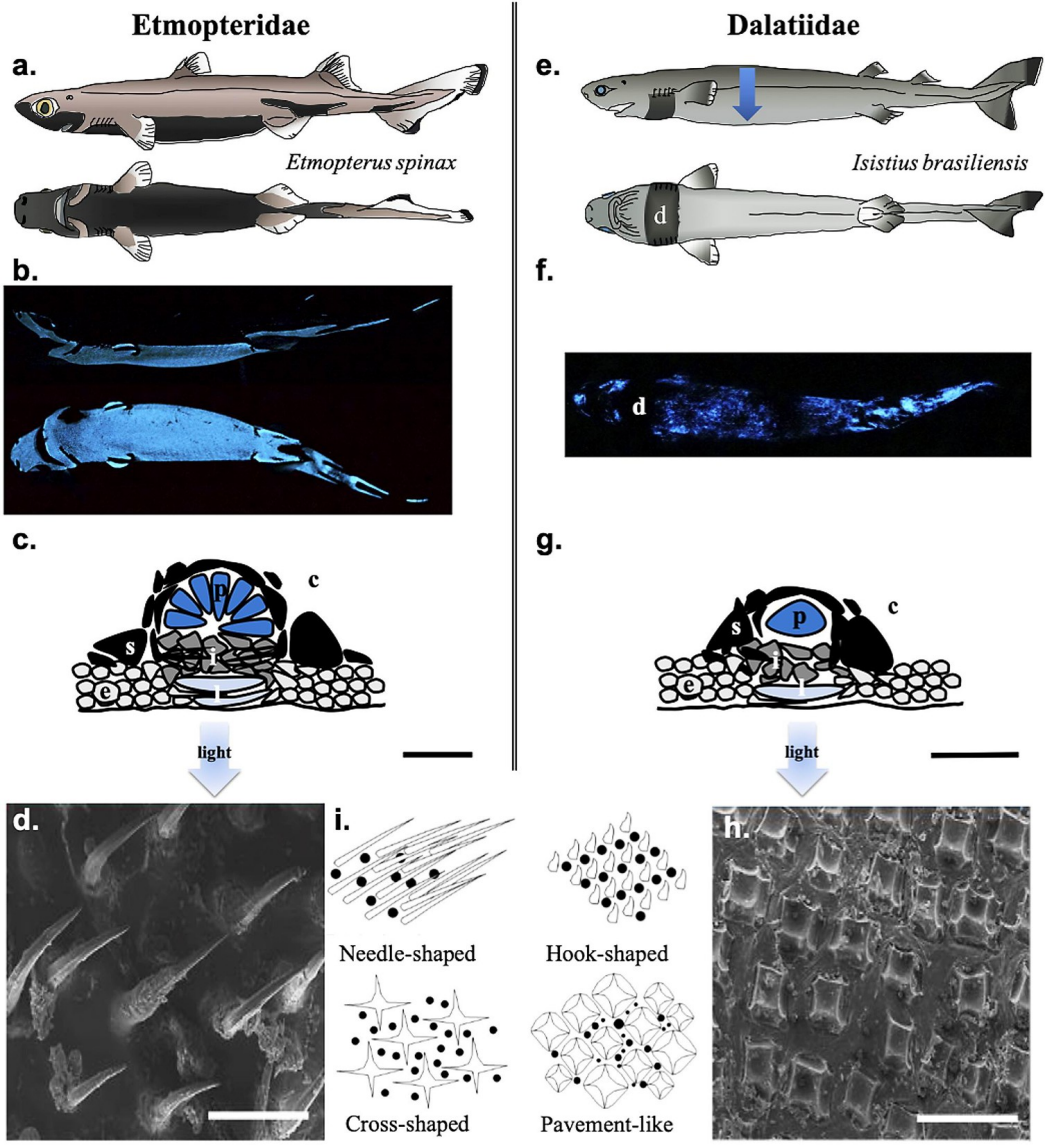
Bioluminescence in Etmopteridae and Dalatiidae. (a). Schematic view of the lateral and ventral luminous pattern of the velvet belly lanternshark (*Etmopterus spinax*), showing specific luminous areas. (b). Spontaneous lateral and ventral luminescence from *E*. *spinax*. (c). Cross-section schematic representation in a typical etmopterid photophore. (d). SEM micrography of *E*. *spinax* needle-shaped denticles. (e) Schematic view of the lateral and ventral luminous pattern of the cookiecutter shark (*Isistius brasiliensis*). The blue arrow illustrates the photophore density gradient from the dorsal (lesser–light blue) to the ventral side (higher–dark blue) of the shark. (f). Spontaneous ventral luminescence from *I*. *brasiliensis*, showing diffused blue luminescence except at the dark collar level. (g). Cross-section schematic representation in a typical dalatiid photophore. (h). SEM micrography of *I*. *brasiliensis* pavement-like denticles. (i). Schematic representation of squamation pattern in luminous sharks (black dots illustrating photophores). Black scale bars, 50 μm; White scale bars, 100 μm. c, connective tissue; d, dark collar; e, epidermis; i, iris-like structure cells; l, lens cells; p, photocyte; s, pigmented sheath.

Bioluminescence, defined as the emission of visible light by a living organism thanks to biochemical reaction, can be derived from either symbiotic bacteria (extrinsic luminescence) or due to own chemical reaction (intrinsic luminescence) [12]. Despite the mention of a luminous species in the Somniosidae family [13], up to now, bioluminescence has been experimentally studied in only two shark families: the kitefin sharks, Dalatiidae, and the lanternsharks, Etmopteridae (Fig 1A, 1B, 1E and 1F) [7, 14, 15]. Shark bioluminescence is intrinsically produced in small specialized organs called photophores (*ca*. 100 μm for Dalatiidae and 150 μm for Etmopteridae) (Fig 1C and 1G) [15, 16]. They possess one or many luminous cells called photocytes (Dalatiidae and Etmopteridae, respectively) embedded in a pigmented sheath and surmounted by one or many lenses allowing light to be focused outward (Fig 1C and 1G) [15–18]. Shark light organs may also possess a guanine crystal-related reflective layer and an iris-like structure serving as light organ shutter (Fig 1C and 1G) [17]. Shark photophores deal with denticles (placoid scales) in terms of space trade-off leading to specific denticle formation which allows light diffusion (Fig 1D, 1H and 1I) [14, 19, 20]. Sharks use mainly bioluminescence for camouflage via counterillumination by ventrally emitting a blue-green light disrupting their shadow (Fig 1B and 1F) [12, 15, 21–23]. While dalatiids present a unique diffuse ventral pattern with a photophore gradient from the flank to the ventral skin body center allowing only counterillumination (Fig 1E–1G) [24], Etmopterids possess a more complex luminous pattern with zones clearly defined for bioluminescence purposes other than counterillumination (Fig 1A–1C). In the lanternshark family, luminescence has been also suggested to be used for intraspecific communication and aposematism [15, 25–30].

**Fig 2:**
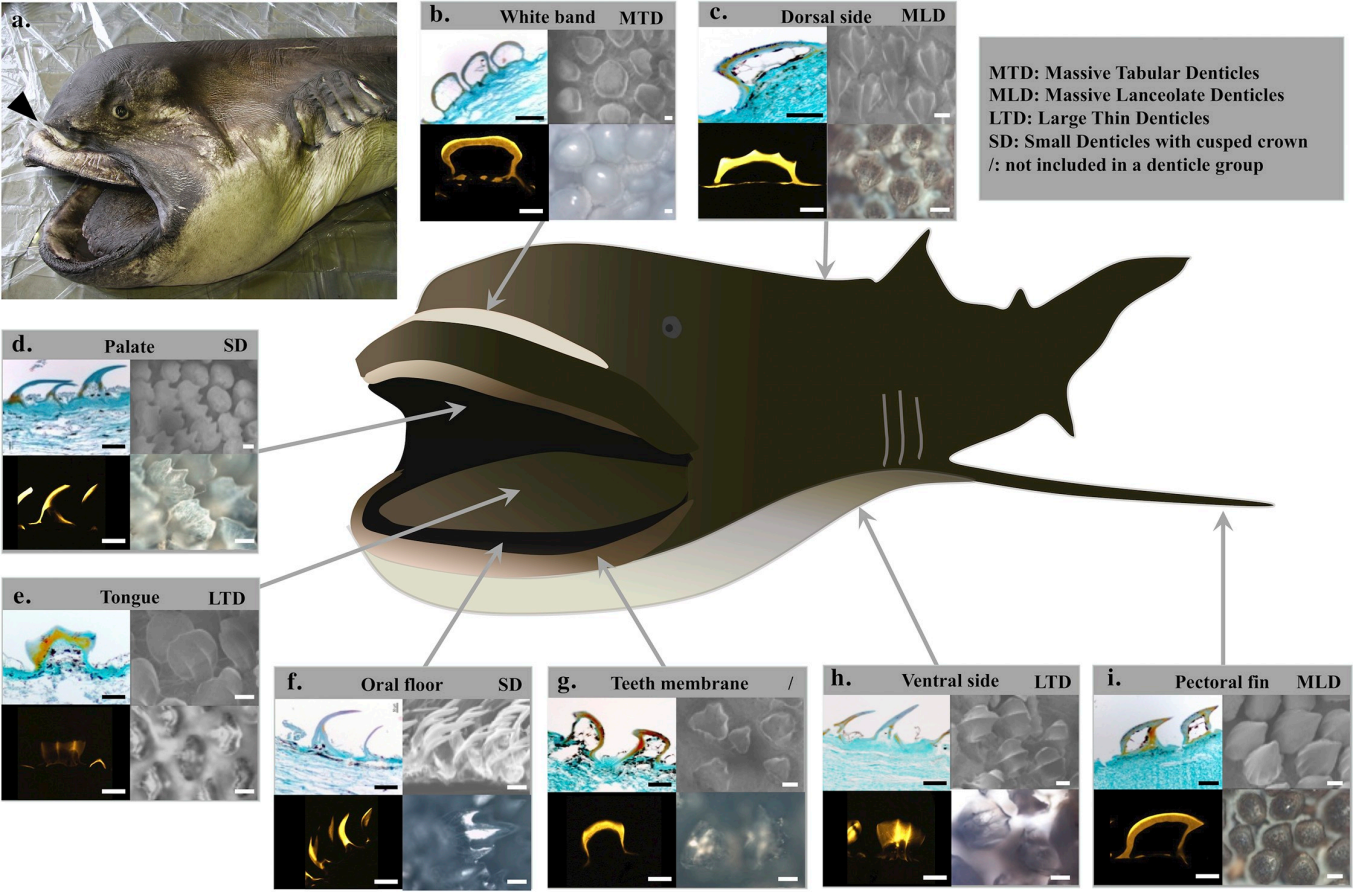
Histological analyses of the megamouth, *Megachasma pelagios*, skin with an emphasis on denticles. (a) Specimen of *M*. *pelagios* (OCA-P 20110301) examined during this work. Arrowhead indicates the white band visible when the jaw is protruded. Histological approaches of the (b) white band, (c) dorsal skin, (d) palate, (e) tongue, (f) oral floor, (g) teeth membrane, (h) ventral skin, (i) pectoral skin. Results are represented for each skin zone: paraffin section (upper left), SEM micrography (upper right), microradiography (lower left), and transmitted light microscope upper view (lower right). Scale bars: 100 μm.

Unlike the little bioluminescent sharks from Dalatiidae and Etmopteridae, mainly measuring less than one meter, the megamouth shark is a large fish that possesses an unusual white band on the upper jaw which contrasts with the black color of the snout and upper jaw [31]. This white band, hidden in a groove between the snout and the jaw, is only visible when the upper jaw is protruded (Fig 2A) [31]. This pattern raises questions about its function, and on a possible bioluminescence use linked to feeding activity [1, 3, 32]. Firstly, Taylor et al. (1983) hypothesized that the white band in *M*. *pelagios* produces light and, in this way, would be used to attract prey [1, 4]. Although frequently cited in the literature [1, 2, 7, 12, 33, 34], no evidence is currently available to confirm this hypothesis. Secondly, Nakaya (2001) assumed that the white band, as any white object, reflects all wavelengths of the visible spectrum [31]. It could then act as a light trap for luring plankton to the proximity of the mouth [31]. Additionally, Nakaya (2001) suggested that this band might be a sort of social signal for conspecific or other species [31]. None of these functions has been proven so far.

The present study aims to test these two hypotheses (*i*.*e*. bioluminescence and reflection) by carrying out an integrative histological analysis of skin samples taken from three megamouth specimens. Epidermis from different locations (*e*.*g*. around the mouth, from the white band, and oral mucosa) were investigated to highlight the presence of symbiotic luminous bacteria and/or light organs as well as the presence of specific denticle patterns allowing light organ development (bioluminescence hypothesis); but also, to detect the presence of specialized light-reflective structures (reflection hypothesis). Results demonstrated (*i*) the absence of either extrinsic or intrinsic luminescence; (*ii*) the high denticle polymorphism around and inside *M*. *pelagios* mouth; (*iii*) the denticle reflective properties (especially at the white band level), supporting the reflector hypothesis possibly involved in a “planktonic luminescence reflection” behavior.

## Material and methods

### Specimen sampling

The three examined *M*. *pelagios* specimens were accidentally caught by local fishermen along the Japanese coast (Table 1). These specimens were sampled at a depth ranging from 0 to 20 meters. Tissue patches of 3 cm^2^ and 0.5 cm thickness were dissected at six different locations around and in the mouth (white band, palate, tongue, oral floor, teeth membrane, back of the throat; Fig 2), observed in transmitted light microscopy and fixed in 4% formalin before use. Besides, epidermal patches from the dorsal, ventral and pectoral fin areas were sampled. As comparative control 3 specimens of a luminous deep-sea shark, *Etmopterus spinax*, were caught in Norway [16]; 3 cm^2^ ventral skin patches were dissected and preserved as *M*. *pelagios* tissues.

**Table 1:** Information on the megamouth shark examined.

*Specimen number*	*Capture location*	*Capture date*	*Sex*	*Size (m)*
OCA-P 20110301*^/^**	Ibaraki prefecture	09/07/2007	Female	3.7
OCA-P 20111217*	Shizuoka prefecture	24/06/2011	Female	5.4
KSW Mp 19–01**	Chiba prefecture	22/05/2017	Female	5.4

* Specimens used for histological analysis.

** Specimens used for fluorescence *in situ* hybridization.

### Bacterial luminescence: Fluorescence *in situ* hybridization

To analyze the potential presence of symbiotic luminescent bacteria in the megamouth shark tissues, fluorescence *in situ* hybridization (FISH) targeting specific bacterial RNA was performed on the suspected luminous zones (*i*.*e*. white band, teeth membrane and back of the throat [1, 2, 4, 7, 12, 33, 34]) from specimens captured in 2011, and 2017 (Table 1). Pieces of *M*. *pelagios* skin tissue previously preserved in formalin were bathed in sterilized phosphate buffer saline (PBS) with increasing concentrations of sucrose (10% for 1 h, 20% for 1 h and 30% overnight). The tissues were then embedded in optimal cutting temperature compound (O.C.T. compound, Tissue-Tek, The Netherlands) and rapidly frozen at -80°C. Cryostat microtome (CM3050 S, Leica, Solms, Germany) was used to obtain 10 μm sections that were laid on coated Superfrost slides and left overnight to dry under sterile conditions. Slides were immersed in successive ethanol solutions (50, 80, and 100%, 3 min each) and left to dry. Then, 10 μL of the hybridization buffer (900 mM sodium chloride, 200 mM Tris/HCl, 40% Formamide and 0.01% SDS in ultrapure water) containing an equimolar mix of three RNA probes (EUB 338 I-cy3; EUB 338 II-cy3 and EUB 338 III-cy3; complementary to a 16S rRNA region conserved for all bacteria) at a final concentration of 5 ng μl^-1^ were applied per slide in the dark at 4°C. As a negative control, a NON EUB-Texas red probe, complementary to the EUB 338 probe, was used to validated nonspecific binding. After the probe application, the slides were incubated in wet conditions at 46°C for 2 h, rinsed and incubated with a washing solution (46 mM NaCl, 20 mM Tris/HCl, 5 mM EDTA and 0.01% SDS in ultrapure water) at 50°C for 25 min. Slides were left to dry, coated with Cityfluor (AF1), and covered with coverslips [16]. All sections were observed with an inverted microscope DMI 6000B (Leica) equipped with a DFC 365 FX camera coupled with LAS AF 3.1.0 software (Leica) to detect any bacterial labeling within the shark epidermis. Positive controls were performed using the extrinsic bioluminescent emitter fish, *Coelorinchus kishinouyei* [16].

### Light organ histology: Paraffin sections

Histological studies (paraffin sections, microradiography, electron microscopy, and spectrophotometry analysis) were performed on two *M*. *pelagios* specimens (captured in 2007 and 2011; Table 1), specimens of *E*. *spinax* were only used for paraffin section in order to provide comparative histology. Pieces of shark skin patches were bathed in a decalcifying solution (OsteoRAL R, Fast Decalcifier for Large Anatomical Specimens, RAL Diagnostics, France) before being embedded in paraffin according to classical histological protocol. Sections of 10 μm thickness were performed with a Reichert microtome and were laid on coated Superfrost slides (Thermo Scientific). Slides were stained with a standard Masson’s Trichrome protocol. Sections were observed with a light microscope (Leitz Diaplan, Germany) equipped with a Nikon Coolpix 950 camera (Nikon, Japan) to highlight the presence of potential light organs. Denticle morphometric measurements (i.e. base and crown widths, length) were also performed on the paraffin histological section of *M*. *pelagios* skin sections (N = 10 per skin zones) using the Image-J software [35].

### Denticle morphology: Microradiography

To analyze the mineralized shark denticle, undecalcified sections of each skin zone (except pieces of skin from the back of the throat which present only a few disparate denticles) were microradiographed. Briefly, skin samples were dehydrated in 100% methanol, bathed in chloroform, rapidly immersed in toluene, and embedded in methyl methacrylate following the manufacturer protocol (Merck, Darmstadt, Germany). After polymerization, transversal sections of 200 μm were obtained using a circular diamond saw (Leica 600; Leica, Wetzlar, Germany) and reduced to a thickness of 80 μm by manual grinding on ground grass plate. Sections were placed on a photosensitive silver halide emulsion (VRP-M Geola; Geola Digital uab, Vilnius, Lithuania), together with a standard aluminum and exposed to X-rays (Machlett tube with tungsten anode and beryllium window, Baltograph, Balteau NDT SA, Hermalle-sous-Argenteau, Belgium) at 17kV, 13.5 mA for 55 min. The irradiated emulsions were bathed 2 min in a developer solution (SM-6 Geola; Geola, Digital uab, Vilnius, Lithuania), briefly bathed in acetic acid 3%, then, fixed for 15 min, rinsed in distilled water, and dried. Sections were observed under polarized light from a Nikon Digital Sight DS-SMc piloted by the software NIS-Elements (Nikon, Tokyo, Japan).

### Denticle morphology: Electron microscopy

Scanning Electron Microscopy (SEM; high-resolution FEG Digital Scanning Microscope 982 Gemini, Leo; Oberkochen, Germany) was used to detail the shape and features of denticles (except pieces of skin from the back of the throat which present only a few disparate denticles). SEM-images were analyzed to describe the denticle density and surface covering percentage. The denticle density and the relative percentage were measured using the Image-J software [35]. At least three measurements for each skin zone were performed.

### Reflective properties: Spectrophotometry

Skin samples from each zone constituted of denticles were analyzed by a spectrophotometer to measure the reflectance and absorbance of the tissue. Spectrophotometric hemispheric measurements were performed with a double-beam spectrophotometer Perkin Elmer Lambda 750S. Skin samples of approximately 1 cm^2^ and 0.5 cm thick from each zone were exposed to a parallel light beam under near-normal incidence.

### Statistical analyses

Statistical analyses were performed on the morphological parameters with JMP® software (JMP®, Version 13. SAS Institute Inc., Cary, NC, 1989–2007.). Since the Gaussian distribution was not obtained, Kruskal-Wallis tests were realized to compare the ratio of the basal and top width for ten denticles measured per zone.

## Results

### Absence of extrinsic or intrinsic luminescent structures

**Fig 3:**
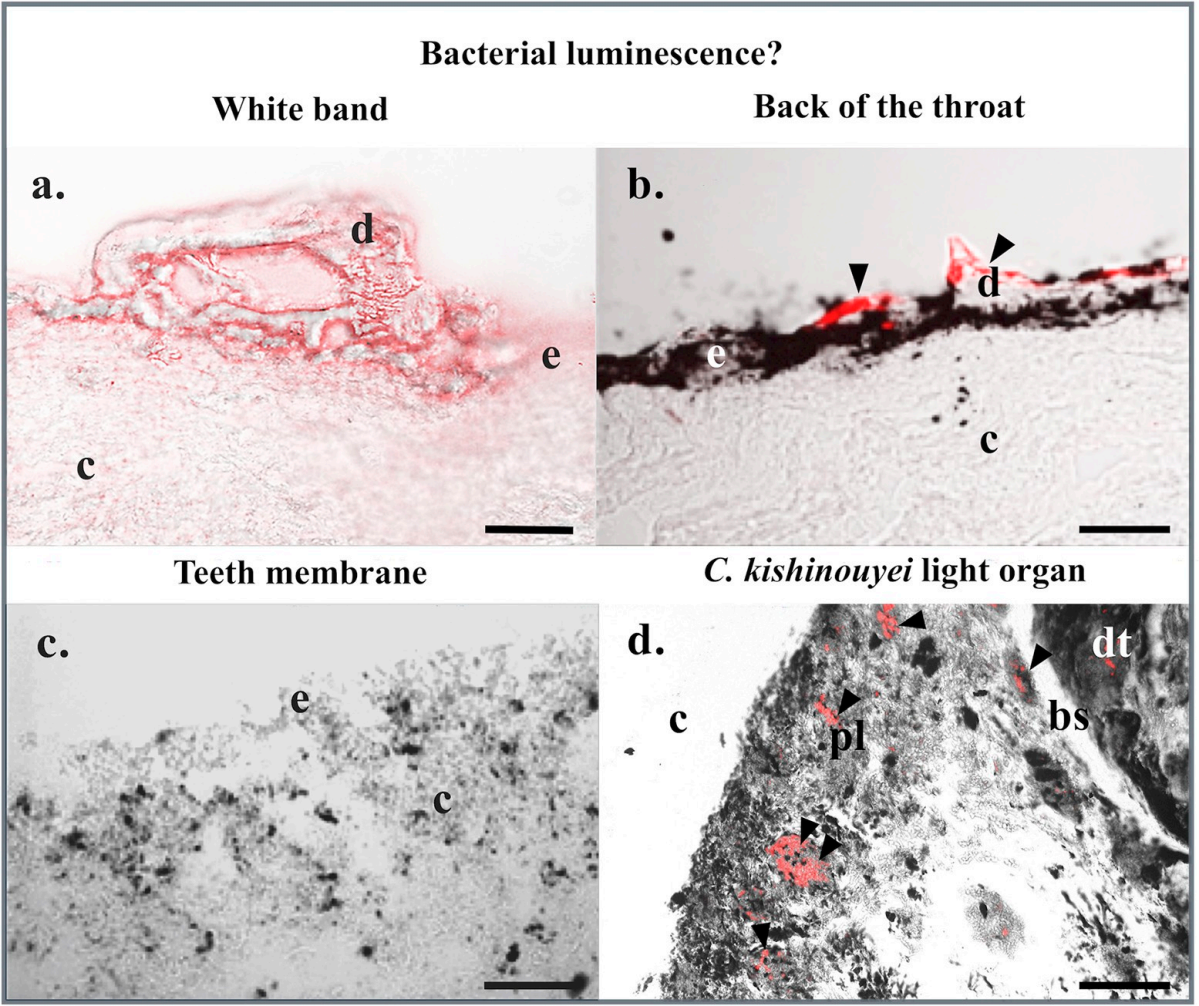
Fluorescence *in situ* hybridization: Bacterial luminescence? Fluorescence *in situ* hybridization on (a) white band, (b) back of the throat, and (c) teeth membrane skin sections of *M*. *pelagios* as well as (d) *C*. *kishinouyei* light organ sections using EUB RNA probes. Black arrowheads indicate bacteria-labeled areas. bs, basal layer of the digestive tract-related tubules; c, connective tissue; d, dermal denticle; dt, digestive tract tubule; e, epidermis; pl, pigmented layer. Scale bars: 100 μm.

**Fig 4:**
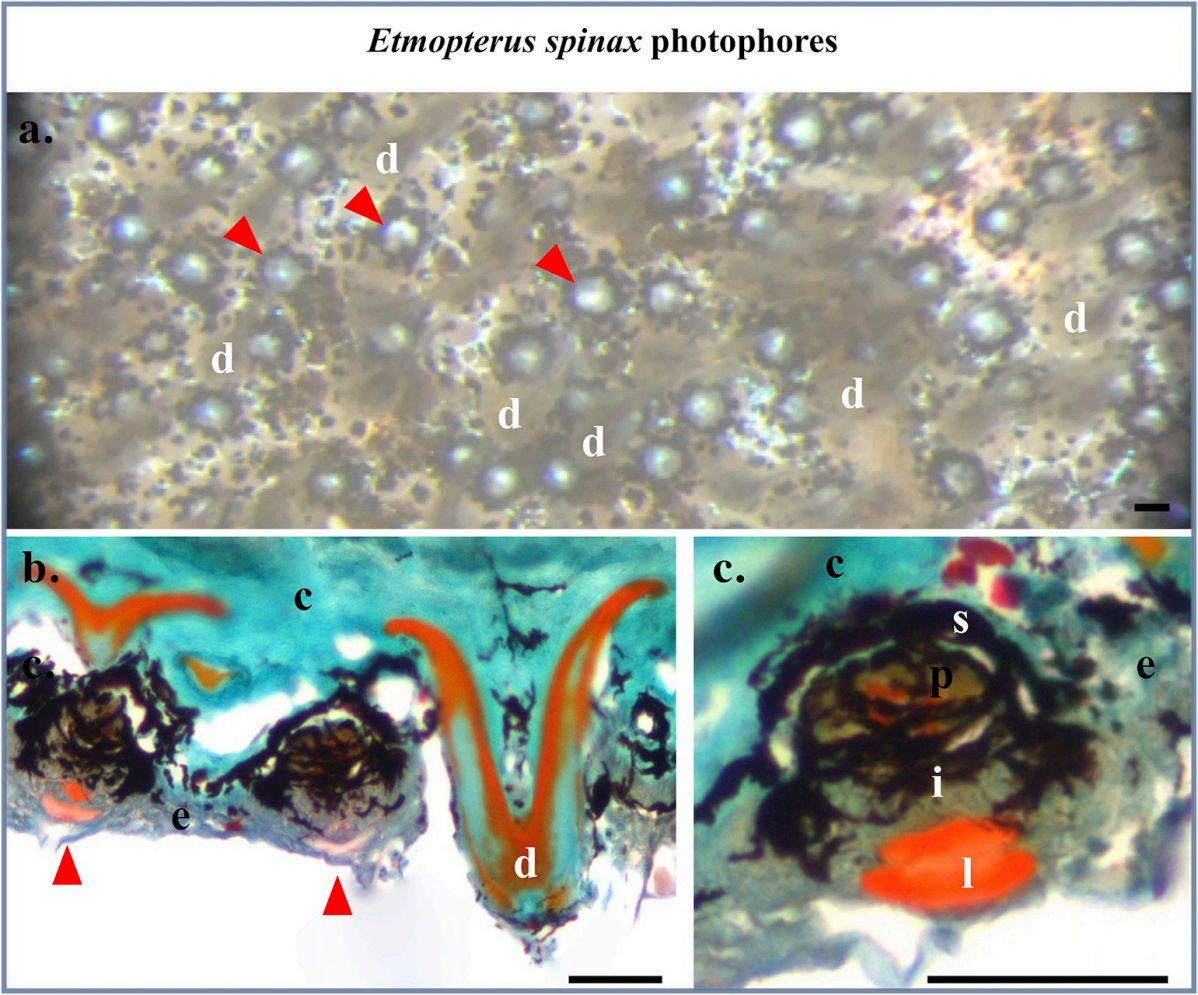
*Etmopterus spinax* photophore histology. (a). Binocular microscope view of the ventral skin of *E*. *spinax*, showing photophores (arrowhead). (b). Masson’s Trichrome staining colored section in the ventral skin of *E*. *spinax* showing photophores (arrowhead). (c) Close-up of the photophore in paraffin cross-section. c, connective tissue; e, epidermis; i, iris-like structure cells; l, lens cells; p, photocytes; s, pigmented sheath. Scale bars: 50 μm.

No bacterial labeling is observed in the megamouth shark white band, membrane, or back of the throat skin patch sections treated with EUB probes (Fig 3A–3C). The signal is absent from all integuments, while a signal is observed at some denticles (Fig 3B; S1 Fig) as observed by Duchatelet et al., 2019 with the same probes for the non luminous shark *Galeus melastomus* [16]. Moreover, negative control performed on *M*. *pelagos* sections using the NON-EUB probe did label only the denticle (S1 Fig). This could be attributed to an aspecific fluorochrome adsorption within the dentine structure of the denticle. Positive control results showed strong labeling at the level of the gastro-intestinal-related light organ of *C*. *kishinouyei* using EUB probes highlighting the presence of bacteria (Fig 3D; S1 Fig), while no labeling was observed using the NON-EUB probe (S1 Fig). Furthermore, none of the megamouth shark skin sections presents typical luminous shark photophore (Figs 2B–2I; 3A–3C), whether it comes from the locations around and within the mouth or the dorsal, pectoral and ventral zones. Conversely, Masson’s Trichome staining of *E*. *spinax* sections reveals the presence of typical lanternshark photophores in the ventral skin (Fig 4). These organs situated within the integument are easily recognizable in histological sections (Fig 4B and 4C): embedded in a dense connective tissue, colored in green, with denticules, colored in orange and green, they harbor, at their base, a hemispherical cup-shaped layer of black-pigmented cells, covering the central photocytes, colored in brown with red nuclear, covered by iris-like structure cells containing black pigments and externally topped by a single or multiple lens cells colored in orange (Fig 4B and 4C).

### Denticle morphologies and morphometrics

Denticles observed in the *M*. *pelagios* skin tissues differ according to their anatomical origin (Fig 2B–2I). Microradiography revealed that enameloid strata are more calcified than dentin in all the sections (Fig 2B–2I). By combining classic histological, microradiography, and SEM analyses, denticle morphology is described and four categories are distinguished: (*i*) the massive tabular denticles; (*ii*) the massive lanceolate denticles; (*iii*) the large thin denticles; (*iv*) the small denticles with a highly cusped crown. Denticles from the back of the throat were found to be disparate and rare (Fig 3B), and are, therefore, not included in this analysis. Denticles from the teeth membrane (also disparate) were not included in the clustering analysis.

**Fig 5:**
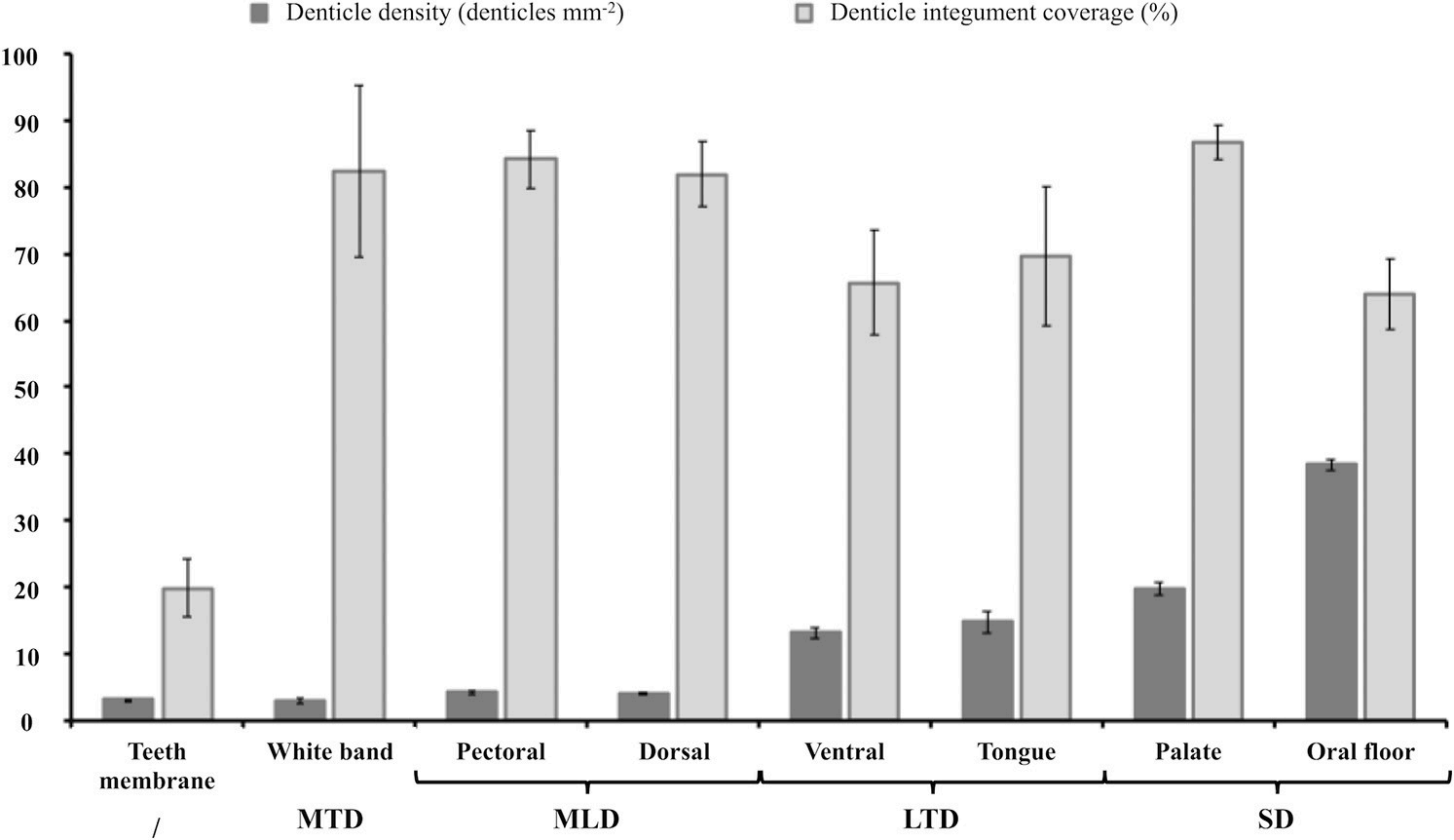
Means of denticle density and integument coverage for each studied *M*. *pelagios* skin area. Denticle groups: MTD, massive tabular denticles; MLD, massive lanceolate denticles; LTD, large thin denticules; SD, small denticles with a cusped crown; /, not included in a denticle group.

Massive Tabular Denticles (MTD) correspond to denticles embedded in the white band. They are massive and broader than the others, with a large base as well as a large crown (Fig 2B; S1 Table). A rounded plateau-like shape is present at the denticle top (Fig 2B). These denticles are highly joined and the edges of one denticle connect almost to the edges of its neighbors (Fig 2B). Because of their large size, they have a low denticle density but a high surface covering percentage (Fig 5; S1 Table). They are the only denticles without any melanophores inside their crown, making them completely white and giving the name to this part of the shark face (Fig 2B). Microradiographies of this denticle group show a high calcification at the crown level compared to the base (Fig 2B).

Massive Lanceolate Denticles (MLD) from dorsal skin and pectoral fins have a triangular crown with a lanceolate apex slightly curved with a peak pointing toward the same direction for all denticles. These denticles are broad and massive, with a large base and crown (Fig 2C and 2I; S1 Table). In the dorsal skin, the denticle crowns possess three well-drawn ridge crests crossing the entire outer surface, one primary and two secondary ridges (Fig 2C), whereas, in the pectoral fins, denticles possess numerous ridges (six to seven) only observed at the first third of the crown length, and not reaching the crown top (Fig 2I). They have a low denticle density but a high surface covering percentage (Fig 5; S1 Table). Like the white band denticles, microradiographies of this denticle group show a highly calcified thick crown compared to the base (Fig 2B, 2C and 2I).

Large Thin Denticles (LTD) are observed in the *M*. *pelagios* ventral skin and on the tongue (Fig 2E and 2H). Their crowns have an intermediate base width and a wide crown top (S1 Table). Denticles from these two regions have slightly curved crowns with three ridges on their convex side (one prominent primary ridge and two secondary) and three cusps (tricuspid). They are in higher density due to their thinness allowing more denticles on the same surface than those from the dorsal and pectoral zones but cover a bit less surface (Fig 5; S1 Table). In the tongue denticle, crowns are trapezed-shaped (Fig 2E), whereas, in the ventral skin, they have a pentagonal shape (Fig 2H). The ridge crests are more sharply sculptured in ventral skin than the tongue denticles, and reach the posterior margin. As well as the ridge crests, the three cusps are more poorly defined with rounded tips and a small cusp length in the tongue (Fig 2E and 2H). Like the two previous denticle groups, microradiographies display highly calcified thick crown compared to the base (Fig 2B, 2C, 2E, 2H and 2I).

Small Denticles with highly Cusped Crown (SD) were found in the palate and oral floor (Fig 2D and 2F). These sharp, blade-like and curved denticles are clustered (Fig 2D and 2F). They are aligned in rows overlapping each other. They are thin at the base and top (S1 Table). These denticles are tricuspid with a primary cusp sharply pointed and two secondary lower cusps but are devoid of ridge crests. Their density is the highest and they cover a higher part of the integument (Fig 5; S1 Table). In the *M*. *pelagios* palate denticles, cusps are less deeply sculptured than denticles from the oral floor. Indeed, cusps lengths are short and slightly cascading and their tips are more rounded.

Denticle morphometric measurements (i.e. base and crown widths, length, crown/base ratio, density, and coverage) are presented in S1 Table. Comparisons between crown/base ratios reveal significant differences between a majority of the studied areas (S1 Table).

### Reflection of the light

Depending on their location, denticles have a different amount of pigments and, therefore, different colors. Denticles are the most opaque and whitest in the white band (Fig 2B). Denticles from the palate and the ventral area present also a white color (Fig 2D and 2H). Embedded in a darkish integument, the oral floor denticles present a pale color without pigment (Fig 2F). Finally, denticles from the dorsal face, the tongue, and pectoral fins are brownish, displaying a lot of dark pigments inside their crowns (Fig 2C, 2E and 2I).

**Fig 6:**
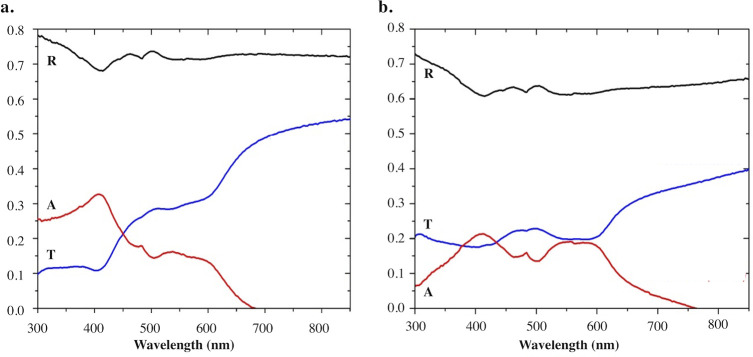
Reflectance and absorbance of the *M*. *pelagios* white band and tongue denticles measured by spectrophotometry. Reflectance (R) and Absorbance (A) measured for (a) the white band and (b) the tongue denticles. Transmittance (T) was evaluated using the following relationship A = 1-T-R.

Fig 6 illustrates the reflectance of the clearest (i.e. white band) and darkest (i.e. tongue) zones. Both tissues reflect at all wavelengths of the visible spectrum (380–800 nm) and part of the ultraviolet (UV) spectrum (300–400 nm). Specifically, the skin tissue from the white band highly reflects light at all wavelengths within the visible spectrum. The reflected light intensity is important in the UV and blue-green wavelengths (i.e. 300 to 500 nm) with a mean value of 72.3% of the incident light (Fig 6A). White band denticles absorb light mainly in the blue spectrum, while the transmittance of light is mainly present between 450 to 800 nm (i.e. between 22 to 54% of the incident light; Fig 6A). Despite being heavily pigmented, the tongue sample showed a mean reflectance of 63.7% of the incident light with highest value in the UV and blue-green wavelengths (Fig 6B). The absorbance of light mainly occurs between 400 and 600 nm, when light is the less transmitted (Fig 6B).

Similarly, skin samples of ventral, dorsal, pectoral, palate, oral floor, and teeth membrane zones also reflect a high percentage of the incident beam (mean reflectance percentages ranging from 60 to 83% for all wavelengths and zones; S1 Fig). All these zones absorb and transmit less than 20% of the incident light between 300 and 600 nm (S1 Fig).

## Discussion

Among the largest shark species on earth, *M*. *pelagios*, the megamouth shark is assumed to be luminous at the upper lip white band [1, 2, 12, 32] or at the back of the throat [32], likely to attract preys. Nakaya (2001) emitted the hypothesis that *M*. *pelagios* is not luminous but instead uses the reflectance of the white band to attract its prey [31]. In the present study, both the absence of labeling for luminous bacteria in the suspected bioluminescent tissues and the absence of any typical light organ (shark photophore) in all studied skin zones, highly suggest that the megamouth shark is not a luminous species. As highlighted in the study of *E*. *spinax* intrinsic luminescence, a weak and strong bacterial labeling is observed for the shark denticle and the extrinsic fish light organ, respectively [16]. Furthermore, even assuming the hypothesis that specific denticles shape and arrangement could be found in luminous shark tissue [13, 14, 36], our results indicated in all skin zones analyzed, except teeth membrane and back of the throat, that the spaces between denticules would be insufficient to allow photophore presence or light propagation. According to our data, the white band integument is completely covered by massive highly calcified tabular denticles with a thick basal anchorage and highly joined by their edges, forming a very cohesive calcified coat which does not allow space for light organs. Although denticle morphology might be important to allow the propagation of light emitted by the photophore [37], the only presence of even bioluminescence-like squamation is not sufficient and does not necessarily mean that the organism is luminous [36]. Our results never highlighted the presence of the “typical luminous squamation” (i.e. needle-, hook-, cross-shaped, or pavement-like [13, 14, 36]) in the megamouth shark. Either intrinsic or extrinsic bioluminescence hypotheses are hence clearly disproved by our data for the megamouth shark.

By using different techniques, a large panel of information for the morphology of denticles (ratio of measures, shape, density, arrangement, surface features, calcification structure, and reflective properties) was obtained. Denticles from the different skin locations were classified into four groups according to morphometric and surface features; hypothetical functions could be found in the literature for each morphology group; although it is difficult to test these functions [13]. According to Raschi and Tabit (1992), each morphology would correspond to a function that ranges in a continuum from skin protection to skin hydrodynamics [20]. More the denticles crowns tend to be heavy, thick and calcified, more they are potentially used to protect the integument against abrasion. Conversely, lighter and thinner they tend to be, more they are potentially shaped to reduce drag (hydrodynamics) [20, 38]. To be efficient, these denticles need to overlap each other forming a continuous surface with ridges [20]. Therefore, Massive Tabular Denticles (MTD) and Massive Lanceolate Denticles (MLD) groups would likely serve to protect tissues from abrasion or ectoparasites such as denticles observed in the central oral cavity of the longtailed carpet shark, *Hemiscyllium ocellatum* [20, 39] or near the snout of the spiny dogfish, *Squalus acanthias* [40]. Denticles from the dorsal side and the pectoral fins possess well-defined ridges on their surface that may also improve hydrodynamism. However, the megamouth shark has a swimming speed of 1.5 to 2.1 km h^-1^ [9] representing for a 5-m-long megamouth shark around 0.1 total body length per second, which is slow for a shark [1]. Therefore, by contrast to highly hydrodynamic denticles, such as those found in fast sharks, which are thinner with ridges and microrelief, *M*. *pelagios* do not present this denticle type. Denticles from the Large Thin Denticles (LTD) group are thinner with ridges alongside their crown and therefore may enhance the hydrodynamic properties or, more particularly, direct the water flow to bring it to sensory organs linked to either motion or food acquisition. That assumption has been already suggested for denticles in other sharks such as those found near sensory organs like ampullae of Lorenzini, pit organs, or taste buds [39, 41–43]. Denticles present on the tongue may also assist in linear water flow inside the mouth and grinding up the plankton and, hence help in the feeding of the megamouth shark, as a plankton filter feeder. Lastly, the Small Denticles (SD) group is assumed to help in the filtration process. These denticles are smaller and thinner than in the other groups and do not possess any ridges but have highly sculpted cusps. Some free-swimming sharks such as Carcharhinid species, using ram ventilation and swimming continuously with their mouth open [44], possess small sculpted cusped denticles which allow to reduce drag on the epithelium of the oropharyngeal cavity [39]. The megamouth shark using a derived ram-filter mode, whereas its mouth is not continuously open, could use its denticles for the same purpose allowing the water flow to pass rapidly inside the mouth when fully open.

Another assumption may be that the denticles play a role in the filtration process via their trident shape and their alignment. Indeed, similar denticles to those observed in the palate, have been observed in the megamouth shark, on gill-raker papillae [1]. They could share their origin and function, such as helping in the filtration and the selection of the food on which the shark is feeding. This hypothesis has been suggested for another filter-feeding shark, the whale shark, that possesses oral denticles that may act as a primitive form of gill-rakers used to feed on plankton [45]. Finally, as suggested by Imms (1905), oropharyngeal denticles may solely be vestigial structures hence not serving anymore for a specific function [46]. The denticle morphologies and morphometrics for the tongue, palate, ventral, dorsal, and pectoral zones described in our study are similar to those described in former studies [1, 47, 48].

The reflection of light (i.e. “reflection hypothesis”), previously suggested by Nakaya (2001) [31], was confirmed by reflectance and transmittance measurements. Older observations of “light emission” in *M*. *pelagios* would be due to the white band denticles highly reflecting the spotlights of observers and divers. Since white band denticles reflect wavelength in the entire visible spectrum, megamouth sharks could use the white band to reflect either downwelling light or luminescence produced by the plankton to attract and feed on it. Although no such *in vivo* behavioral clues were presented here, the large amount of luminous prey in *M*. *pelagios* stomach contents (*i*.*e*. *Euphausia pacifica*, *Nematoscelis difficilis*, *Thysanopoda* genus, *Atolla* genus, copepods [8, 49, 50]), and positive phototactism of some of them [51–53], adds support to this assumption. This strategy consisting to use bioluminescence of other organisms to attract prey is similar to the one suggested for the sperm whale, *Physeter microcephalus*. This cetacean is thought to use the bioluminescence emitted by deep-sea planktonic organisms to attract predatory squids to its mouth [54]. Field observation of the *M*. *pelagios* feeding behavior at depth could help to better understand the white band function and importance for this elusive organism.

## Conclusion

Based on bacterial detection as well as histological and reflectance analyses, our results strongly support the statement that the megamouth shark, *M*. *pelagios*, is not a luminous shark species. Histological analyses of denticles in the epidermal tissues around and within the mouth revealed a large variety of denticle morphotypes, which ranged into four groups. Hypothetical ecological functions for each denticle morphotype were suggested according to their morphology.

The strong reflectance of the denticles in the white band is most likely responsible for the "glow" of the megamouth shark, due to the reflection of the descending light or the light produced by their planktonic prey or even the diving light used by divers who have been lucky enough to see a live specimen swimming at dusk or dawn.

## Supporting information

S1 Table Morphometric parameters of the *Megachasma pelagios* studied placoid scales. (a) Mean base width, crown width, length and crown/base width ratio for the different studied placoid scale zones. (b) Denticle density and percentage of integument coverage for the different studied placoid scale zones. Values are mean ± s.e.m. (c) Two by two Krustal-Wallis test comparisons between crown/base ratios. * indicate significant differences.
(PDF)

S1 Fig Fluorescence *in situ* hybridization: NON-EUB control. Fluorescence *in situ* hybridization on (a) back of the throat section treated with EUB probes, (b) back of the throat section treated with the NON-EUB-Texas red probe. Both sections present a labeling at the placoid scale level (red arrowhead). Fluorescence *in situ* hybridization on the extrinsic bioluminescent fish, *Coelorinchus kishinouyei* gastro-intestinal gland section (c) with EUB probes, presenting a labeling within the gland tubule (red arrowhead), and (d) with NON-EUB-Texas red probe. c, connective tissue; d, dermal denticle; e, epidermis; pl, pigmented layer; t, tubule. Scale bar: 100 μm.
(PDF)

S2 Fig Reflectance, absorbance and transmittance of *M*. *pelagios* denticles from different tissues measured by spectrophotometry. Reflectance, absorbance and transmittance for (a) ventral skin, (b) dorsal skin, (c) pectoral fin skin, (d) palate, (e) oral floor, and (f) membrane zone denticles. Tissues reflect at all wavelength of the visible spectrum and part of the ultraviolet spectrum (300–400 nm). Black, red and blue curves represent the reflectance (R), absorbance (A) and transmittance (T), respectively.
(PDF)

S1 File Excel file with raw data on the placoid scale morphometrics (sheet 1), density and coverage measurements (sheet 2), and spectrometry analyses (sheet 3). (XLSX)
