# Supplementary material for: The megamouth shark, Megachasma pelagios, is not a luminous species
Source: PLoS One. 2020 Nov 25;15(11):e0242196. doi: 10.1371/journal.pone.0242196 (PMC7688146; doi:10.1371/journal.pone.0242196)
Supplement: S1 Table — (a) Mean base width, crown width, length and crown/base width ratio for the different studied placoid scale zones. (b) Denticle density and percentage of integument coverage for the different studied placoid scale zones. Values are mean ± s.e.m. (c) Two by two Krustal-Wallis test comparisons between crown/base ratios. * indicate significant differences. (PDF) [file pone.0242196.s001.pdf]

The megamouth shark, *Megachasma pelagios* is not a luminous species
PloS One
L. Duchatelet, V. Moris, T. Tomita, J. Mahillon, K. Sato, C. Behets, J. Mallefet
Corresponding authors: L. Duchatelet: laurent.duchatelet@uclouvain.be

---

S1 Table. Morphometric parameters of the *Megachasma pelagios* studied placoid scales. (a) Mean base width, crown width, length and crown/base width ratio for the different studied placoid scale zones. (b) Denticle density and percentage of integument coverage for the different studied placoid scale zones. Values are mean ± s.e.m. (c) Two by two Krustal-Wallis test comparisons between crown/base ratios. * indicate significant differences.

(a)

Group	Zone	Base width (µm)	Crown width (µm)	Length (µm)	Crown/Base width ratio (%)
/	Teeth membrane	163.02 ± 35.12	116.48 ± 27.76	238.07 ± 48.07	71.96 ± 13.71
MTD	White band	358.83 ± 72.72	339.74 ± 54.94	240.31 ± 31.09	95.63 ± 6.93
MLD	Dorsal skin	443.86 ± 74.23	271.82 ± 67.15	170.77 ± 35.04	60.80 ± 7.52
	Pectoral skin	448.31 ± 77.14	252.34 ± 47.04	307.64 ± 35.82	57.25 ± 11.73
LTD	Ventral skin	153.86 ± 33.99	198.32 ± 24.92	214.21 ± 23.55	30.01 ± 5.93
	Tongue	183.40 ± 26.89	239.81 ± 38.18	173.17 ± 36.65	131.20 ± 13.85
SD	Palate	95.92 ± 17.02	18.68 ± 7.07	166.87 ± 54.34	18.42 ± 4.78
	Oral floor	81.83 ± 33.79	17.40 ± 4.16	172.93 ± 31.12	26.06 ± 15.96

(b)

Group	Zone	Density (denticle mm^{-2})	Coverage (%)
/	Teeth membrane	3.11 ± 0.06	19.87 ± 4.44
MTD	White band	2.91 ± 0.36	82.37 ± 12.35
MLD	Dorsal skin	3.99 ± 0.21	81.89 ± 4.90
	Pectoral skin	4.16 ± 0.35	84.21 ± 4.31
LTD	Ventral skin	13.11 ± 0.88	65.66 ± 7.85
	Tongue	14.76 ± 1.63	69.63 ± 10.37
SD	Palate	19.68 ± 0.89	86.81 ± 2.57
	Oral floor	38.35 ± 0.75	63.87 ± 5.23

(c)

Zone	Zone	P-value	Zone	Zone	P-value
Ventral skin	White band	0.0002*	Pectoral skin	Tongue	0.0002*
Ventral skin	Tongue	0.0002*	Pectoral skin	Teeth membrane	0.0640
Ventral skin	Teeth membrane	0.0002*	Pectoral skin	Palate	0.0002*
Ventral skin	Palate	0.0013*	Pectoral skin	Oral floor	0.0017*
Ventral skin	Oral floor	0.0757	Oral floor	White band	0.0002*
Ventral skin	Dorsal skin	0.0002*	Oral floor	Tongue	0.0002*
Ventral skin	Pectoral skin	0.0002*	Oral floor	Teeth membrane	0.0008*
Dorsal skin	White band	0.0002*	Oral floor	Palate	0.3447
Dorsal skin	Tongue	0.0002*	Teeth membrane	White band	0.0010*
Dorsal skin	Teeth membrane	0.0757	Teeth membrane	Tongue	0.0002*
Dorsal skin	Palate	0.0002*	Teeth membrane	Palate	0.0002*
Dorsal skin	Oral floor	0.0017*	Palate	White band	0.0002*
Dorsal skin	Pectoral skin	0.6232	Palate	Tongue	0.0002*
Pectoral skin	White band	0.0002*	White band	Tongue	0.0002*
