# Supplementary material for: The megamouth shark, Megachasma pelagios, is not a luminous species
Source: PLoS One. 2020 Nov 25;15(11):e0242196. doi: 10.1371/journal.pone.0242196 (PMC7688146; doi:10.1371/journal.pone.0242196)

The megamouth shark, *Megachasma pelagios* is not a luminous species

PloS One

L. Duchatelet, V. Moris, T. Tomita, J. Mahillon, K. Sato, C. Behets, J. Mallefet
Corresponding authors: L. Duchatelet: laurent.duchatelet@uclouvain.be

---

S1 Fig. Fluorescence *in situ* hybridization: NON-EUB control. Fluorescence *in situ* hybridization on (a) back of the throat section treated with EUB probes, (b) back of the throat section treated with the NON-EUB-texas red probe. Both sections present a labeling at the placoid scale level (red arrowhead). Fluorescence *in situ* hybridization on the extrinsic bioluminescent fish, *Coelorinchus kishinouyei* gastro-intestinal gland section (c) with EUB probes, presenting a labeling within the gland tubule (red arrowhead), and (d) with NON-EUB-texas red probe. c, connective tissue; d, dermal denticle; e, epidermis; pl, pigmented layer; t, tubule. Scale bar: 100 µm.

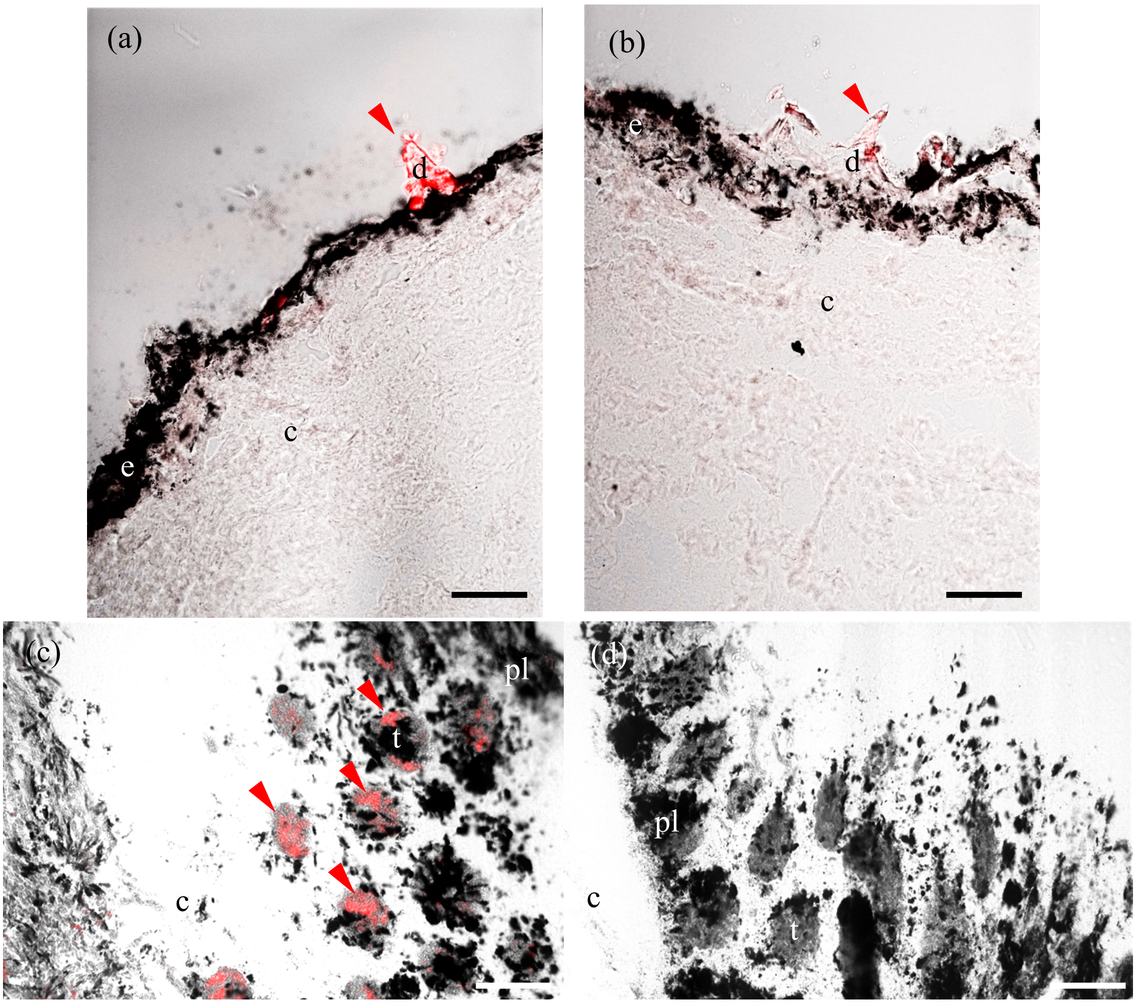

Supplement: S1 Fig — Fluorescence in situ hybridization on (a) back of the throat section treated with EUB probes, (b) back of the throat section treated with the NON-EUB-Texas red probe. Both sections present a labeling at the placoid scale level (red arrowhead). Fluorescence in situ hybridization on the extrinsic bioluminescent fish, Coelorinchus kishinouyei gastro-intestinal gland section (c) with EUB probes, presenting a labeling within the gland tubule (red arrowhead), and (d) with NON-EUB-Texas red probe. c, connective tissue; d, dermal denticle; e, epidermis; pl, pigmented layer; t, tubule. Scale bar: 100 μm. (PDF) [file pone.0242196.s002.pdf]
