# Supplementary material for: The megamouth shark, Megachasma pelagios, is not a luminous species
Source: PLoS One. 2020 Nov 25;15(11):e0242196. doi: 10.1371/journal.pone.0242196 (PMC7688146; doi:10.1371/journal.pone.0242196)
Supplement: S2 Fig — Reflectance, absorbance and transmittance for (a) ventral skin, (b) dorsal skin, (c) pectoral fin skin, (d) palate, (e) oral floor, and (f) membrane zone denticles. Tissues reflect at all wavelength of the visible spectrum and part of the ultraviolet spectrum (300–400 nm). Black, red and blue curves represent the reflectance (R), absorbance (A) and transmittance (T), respectively. (PDF) [file pone.0242196.s003.pdf]

The megamouth shark, *Megachasma pelagios* is not a luminous species

PloS One

L. Duchatelet, V. Moris, T. Tomita, J. Mahillon, K. Sato, C. Behets, J. Mallefet
Corresponding authors: L. Duchatelet: laurent.duchatelet@uclouvain.be

---

S2 Fig. Reflectance, absorbance and transmittance of *M. pelagios* placoid scales from different tissues measured by spectrophotometry. Reflectance, absorbance and transmittance for (a) ventral skin, (b) dorsal skin, (c) pectoral fin skin, (d) palate, (e) oral floor, and (f) teeth membrane zone placoid scales. Tissues reflect at all wavelength of the visible spectrum and part of the ultraviolet spectrum (300-400 nm). Black, red and blue curves represent the reflectance (R), absorbance (A) and transmittance (T), respectively.

(a)

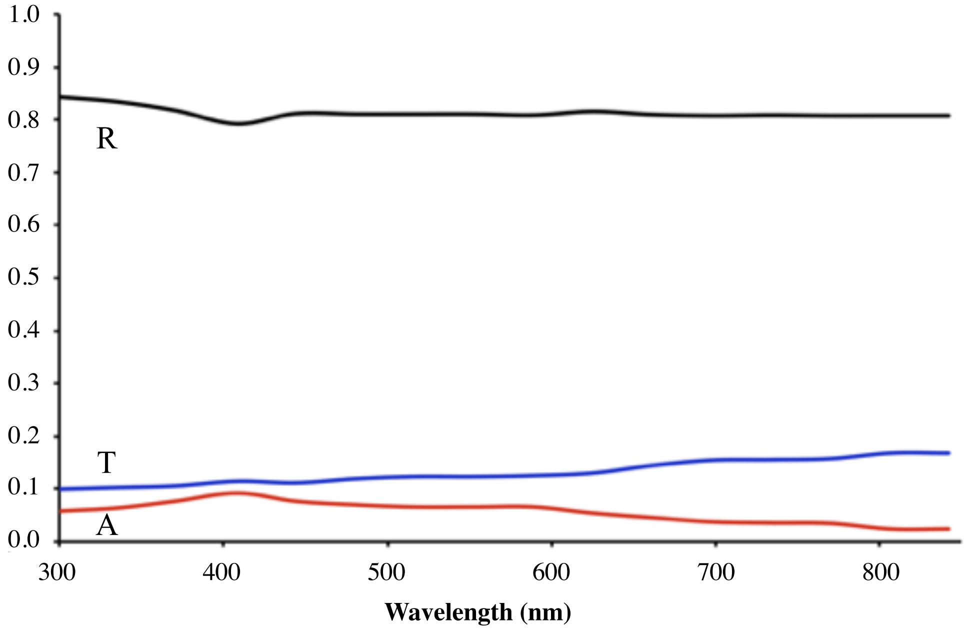

(b)

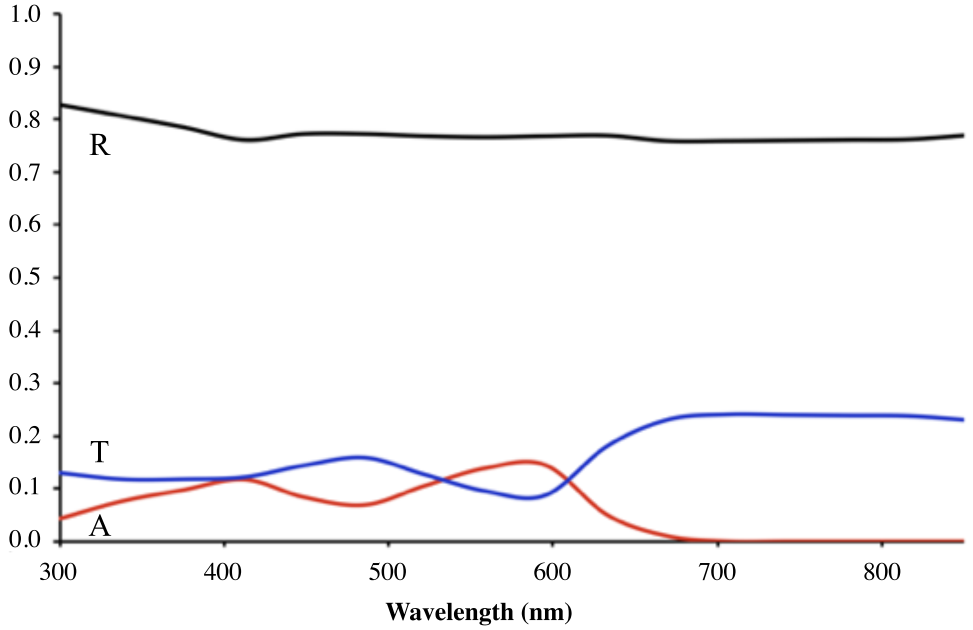

(c)

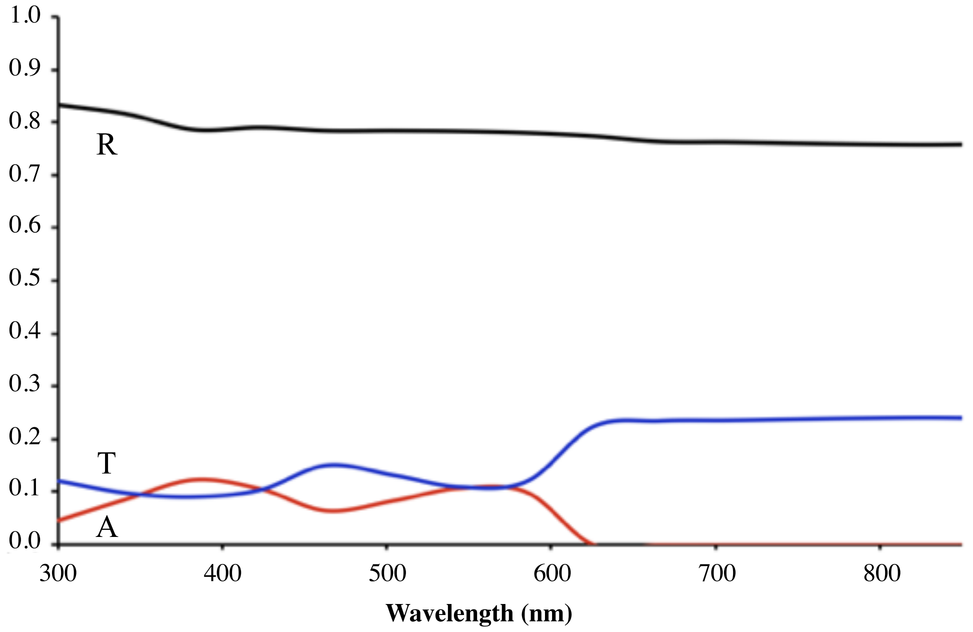
1.0
0.9
0.8
0.7
0.6
0.5
0.4
0.3
0.2
0.1
0.0
R
T
A
300
400
500
600
700
800
Wavelength (nm)

(d)

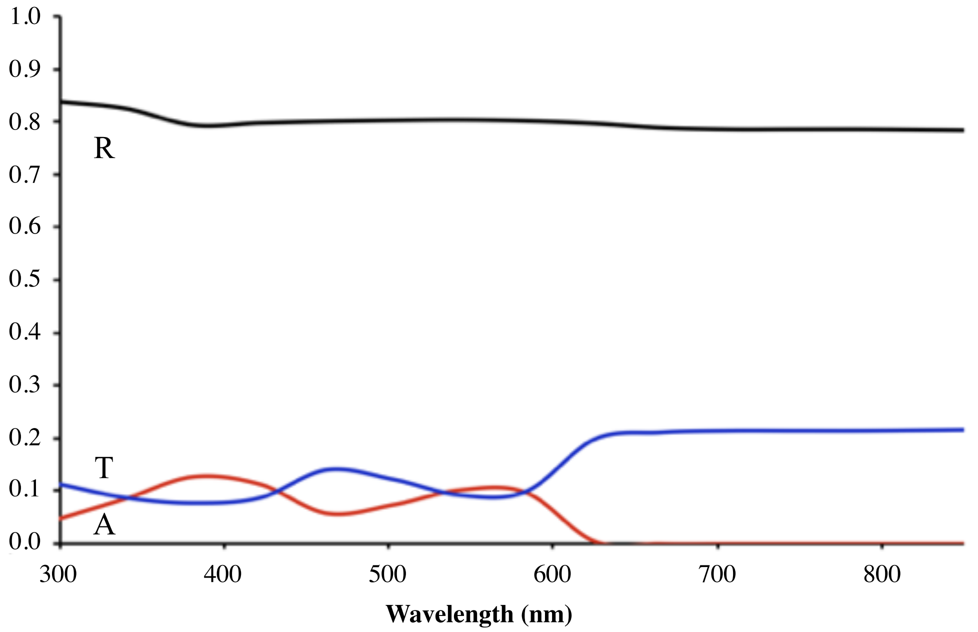
1.0
0.9
0.8
0.7
0.6
0.5
0.4
0.3
0.2
0.1
0.0
R
T
A
300
400
500
600
700
800
Wavelength (nm)

(e)

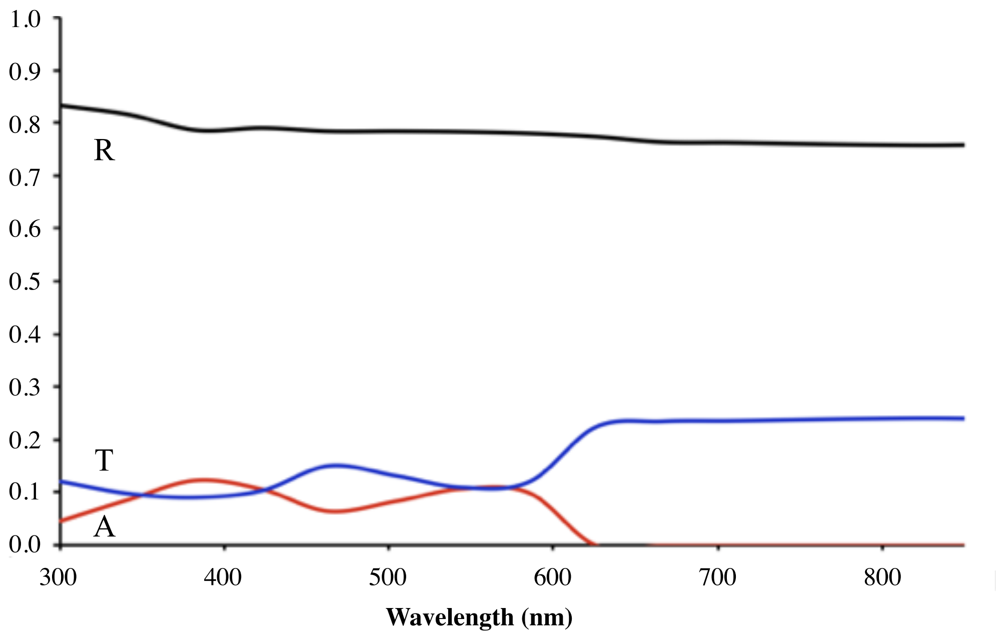
1.0
0.9
0.8
0.7
0.6
0.5
0.4
0.3
0.2
0.1
0.0
R
T
A
300
400
500
600
700
800
Wavelength (nm)

(f)

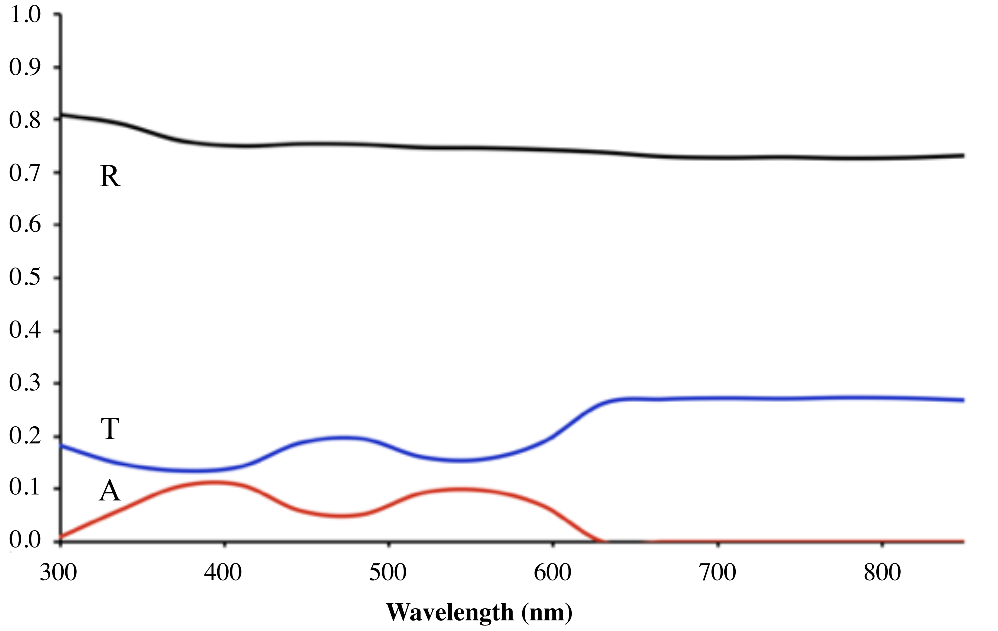
1.0
0.9
0.8
0.7
0.6
0.5
0.4
0.3
0.2
0.1
0.0
R
T
A
300
400
500
600
700
800
Wavelength (nm)
